# Health-related quality of life after direct endovascular thrombectomy and bridging therapy: findings of DIRECT-MT trial

**DOI:** 10.3389/fneur.2026.1768325

**Published:** 2026-05-25

**Authors:** Renjun Gu, Meihua Huyan, Hongjian Zhang, Fang Shen, Hongjian Shen, Zifu Li, Pengfei Xing, Weilong Hua, Yongxin Zhang, Jianmin Liu, Pengfei Yang, Lei Zhang, Yongwei Zhang

**Affiliations:** 1 Neurovascular Center, Changhai Hospital, Naval Medical University, Shanghai, China; 2 Key Laboratory of Molecular Neurobiology of Ministry of Education, Naval Medical University, Shanghai, China; 3 Oriental Pan-Vascular Devices Innovation College, University of Shanghai for Science and Technology, Shanghai, China; 4 Changhai Clinical Research Unit, Naval Medical University, Shanghai, China

**Keywords:** DIRECT-MT trial, endovascular thrombectomy, health-related quality of life, intravenous alteplase, ischemic stroke

## Abstract

**Background:**

Endovascular thrombectomy (EVT) alone is noninferior to bridging therapy for functional outcomes after large-vessel occlusion stroke in patients directly admitted to comprehensive stroke centers, but effects on health-related quality of life (HRQoL) are uncertain. We compared HRQoL between treatment strategies and assessed effect modification by baseline severity and functional outcomes strata.

**Methods:**

This secondary analysis of the DIRECT-MT trial compared EVT alone with bridging therapy. HRQoL at 90 days was measured by EQ-5D-5L index. Quantile regression tested treatment effects on median EQ-5D with interaction terms for baseline NIHSS categories (0–15, 16–20, 21–42) and exploratory 90-day BI and mRS strata. EQ-5D dimensions were analyzed using logistic regression for symptom-free status.

**Results:**

Among 656 patients (327 EVT alone; 329 bridging), overall HRQoL among 90-day survivors was similar between groups (median EQ-5D 0.84 vs. 0.85). Baseline stroke severity modified the treatment-EQ-5D association (*P* for interaction = 0.038), with bridging therapy associated with higher EQ-5D among patients with moderate-to-severe stroke (NIHSS 16–20) and severe stroke (NIHSS 21–42). In exploratory post-randomization analyses, bridging therapy was favored among patients with near-complete independence (BI 91–100; coefficient −0.056; 95% CI −0.112 to −0.001), whereas EVT alone was associated with lower EQ-5D among those with a favorable functional outcome (mRS 0–2; coefficient −0.026; 95% CI −0.044 to −0.009). In dimension-level analyses, EVT alone was associated with lower odds of symptom-free Mobility and Usual activities in the near-independent group (BI 91–100), and a treatment-by-baseline severity interaction was observed for Anxiety/depression (*P* for interaction = 0.018).

**Conclusions:**

HRQoL at 90 days was comparable overall between strategies, but differed by baseline stroke severity. Exploratory analyses suggested additional heterogeneity across achieved functional states and EQ-5D dimensions, supporting further evaluation of baseline severity-guided approaches to optimize patient-centered outcomes.

## Introduction

Acute ischemic stroke due to large-vessel occlusion is a leading cause of morbidity and mortality worldwide, for which rapid reperfusion is the cornerstone of treatment (1). Endovascular thrombectomy (EVT) is now the standard of care for eligible patients with anterior circulation large-vessel occlusion, providing substantial functional benefit within appropriate time windows (2–4). Intravenous alteplase has traditionally been administered before EVT, and bridging therapy has long been recommended by guidelines when feasible (1, 4, 5).

However, there is uncertainty regarding the role of alteplase before and during thrombectomy in patients with ischemic stroke. Recent randomized trials, including the DIRECT-MT trial, have questioned the added value of bridging therapy (2, 6). DIRECT-MT and other studies have demonstrated the non-inferiority of EVT alone compared with combination therapy for functional independence, as measured by the modified Rankin Scale (mRS), at 90 days in patients directly admitted to comprehensive stroke centers. However, the broader impact of these treatment strategies on patient-centered outcomes, particularly health-related quality of life (HRQoL), remains uncertain (6). Functional scales such as mRS and Barthel Index (BI) provide critical measures of disability and independence, but they may not fully capture patients' perceived well-being across domains such as mobility, self-care, pain, and mental health (7). The EuroQol-5 Dimensions (EQ-5D) instrument offers a standardized measure of HRQoL, enabling assessment of treatment effects beyond conventional functional outcomes (8).

Importantly, treatment effects on HRQoL may vary across patient subgroups. Baseline stroke severity, commonly assessed by the National Institutes of Health Stroke Scale (NIHSS), is available before treatment and may inform clinically actionable heterogeneity of treatment effects. In contrast, functional status measured at 90 days (e.g., BI and mRS) reflects achieved recovery and can help describe how HRQoL differences manifest across follow-up functional states, although such post-treatment strata are not available for treatment selection at baseline.

Therefore, in this secondary analysis of the DIRECT-MT trial, we evaluated HRQoL at 90 days using the EQ-5D index in patients randomized to EVT alone versus bridging therapy. Specifically, we aimed to: (1) compare overall HRQoL outcomes between treatment strategies; (2) assess whether baseline stroke severity modifies the association between treatment strategy and HRQoL; (3) explore, as post-randomization descriptive analyses, heterogeneity of HRQoL differences across functional outcome strata defined by 90-day BI and mRS; and (4) examine EQ-5D dimension-specific outcomes to identify domains influenced by treatment assignment and potential effect modification across baseline stroke severity and functional outcomes strata.

## Methods

### Study design and population

This is a secondary analysis of the DIRECT-MT randomized clinical trial, which was an investigator-initiated, multicenter, prospective, randomized, open-label trial with blinded outcome assessment involving patients with acute ischemic stroke who were eligible both to receive intravenous alteplase and to undergo EVT (**Registration:** URL: clinicaltrials.gov, Identifier: NCT03469206) (6). It aimed to determine whether EVT alone would be non-inferior to combined treatment with EVT preceded by intravenous alteplase in patients who had acute ischemic stroke with large-vessel occlusion in the anterior circulation. Details of the trial protocol, eligibility criteria, and primary results have been reported previously (9).

Patients underwent randomization centrally to undergo EVT alone or to receive combined treatment with intravenous alteplase (at a dose of 0.9 mg per kilogram of body weight) before EVT. The ethics board at each site approved the trial. Informed consent was obtained from patients or their surrogates directly or using a deferral of consent, followed by regained capacity consent for ongoing participation process at sites where that was permitted.

### Outcome assessment

The primary HRQoL outcome was the EQ-5D index at 90 days (within a window of ±14 days) after randomization, derived from the EuroQol 5-Dimension 5-Level questionnaire (EQ-5D-5L) questionnaire using the Chinese value set. The EQ-5D includes five dimensions: Mobility, Self-care, Usual activities, Pain/discomfort, and Anxiety/depression. Each dimension has five response levels, 1 (no problems/symptom-free), 2 (slight problems), 3 (moderate problems), 4 (severe problems) and 5 (extreme problems), allowing for the description of 3,125 unique health states. Domains are combined to create an index value that ranges from −0.39 (state worse than dead), through 0 (equal to dead), to 1 (perfect health) (10).

### Effect modifiers

#### Effect modification by baseline stroke severity

To assess clinically actionable effect modification, baseline stroke severity was evaluated using NIHSS categories defined *a priori* as mild-to-moderate (0–15), moderate-to-severe (16–20), and severe (21–42) stroke (11, 12). These cutoffs reflect clinically meaningful severity strata and were chosen to ensure adequate sample size within each category. Interaction terms of treatment and NIHSS category were incorporated into the quantile regression models assessing EQ-5D index at 90 days.

#### Exploratory post-randomization stratification by 90-day functional status

Functional outcome measures assessed at 90 days (within ±14 days after randomization), namely the BI and mRS, were used to define exploratory post-randomization strata for descriptive and interaction analyses of EQ-5D (13). These 90-day BI/mRS strata were not intended to be used as adjustment covariates; rather, they were used to describe how HRQoL differences manifest across achieved functional states at follow-up. The BI is a widely used tool to assess a patient's independence in performing basic activities of daily living. The total score on the BI ranges from 0 to 100, with higher scores indicating greater independence. BI scores were categorized into four ranges: 0–20 (complete dependence), 21–60 (severe dependence), 61–90 (moderate dependence), and 91–100 (slight dependence to independence) (14, 15). The mRS was dichotomized into 0–2 (favorable outcome) versus 3–5 (unfavorable outcome), with a score of 6 indicating death (16, 17).

### Statistical analysis

According to distributional properties of the data, baseline characteristics were reported as absolute and relative frequencies, mean ± standard deviation (SD), or median with interquartile range (IQR). Subgroups were compared according to the descriptive statistics above using *t*-test, Mann–Whitney test, Fisher's exact test and Yates's correction for continuity of Chi-square Test.

Median EQ-5D values with IQR were reported overall and within subgroups. Differences between patients undergoing EVT alone and receiving combined treatment with intravenous alteplase before EVT were explored descriptively. To formally assess treatment effects and effect modification, quantile regression models were fitted with EQ-5D index as the dependent variable and treatment group as the primary independent variable. Effect modification was evaluated by including treatment-by-subgroup interaction terms for (i) baseline NIHSS categories (primary, pre-randomization interaction analysis) and (ii) exploratory post-randomization strata defined by 90-day BI or mRS. Models were adjusted for prespecified baseline covariates, including demographics, medical history, laboratory values. Baseline NIHSS was summarized as a continuous variable in baseline tables but categorized for interaction analyses to facilitate clinical interpretability of subgroup effects. In a first sensitivity analysis, we used multiple imputation with truncated regression (for EQ-5D index maximum range −0.39 to 1.00) to impute the missing EQ-5D index, including the same covariates in the imputation model as above. In a second sensitivity analysis, we excluded those with death at 90 days. Lastly, we applied quantile regression to the 25th and 75th percentiles rather than the median EQ-5D to confirm the relationships were similar across the distribution of EQ-5D index.

Among 90-day survivors, this study then calculated the percent of individuals within each subdomain of EQ-5D (Mobility, Self-care, Usual activities, Pain/discomfort, and Anxiety/depression) stratified by treatment groups. Due to the large number in category 1 (symptom-free), we used logistic regression to further evaluate the association between direct EVT and achieving symptom-free status, adjusting for the same prespecified baseline covariates as in the primary analyses. We also assessed effect modification by baseline NIHSS categories for each EQ-5D dimension using treatment-by-baseline NIHSS interaction terms, and -by-functional status (BI/mRS) at 90 days. In this study, deaths were assigned a value of 0 for the primary analysis. Sensitivity analyses were conducted imputing missing values with the multiple imputation with truncated regression, or excluding deaths altogether.

All analyses were performed using SPSS 26.0 and R 4.4.2. All P-values were two-sided, and conventional levels of significance (α=0.05) were used for interpretation.

## Results

**Figure 1:**
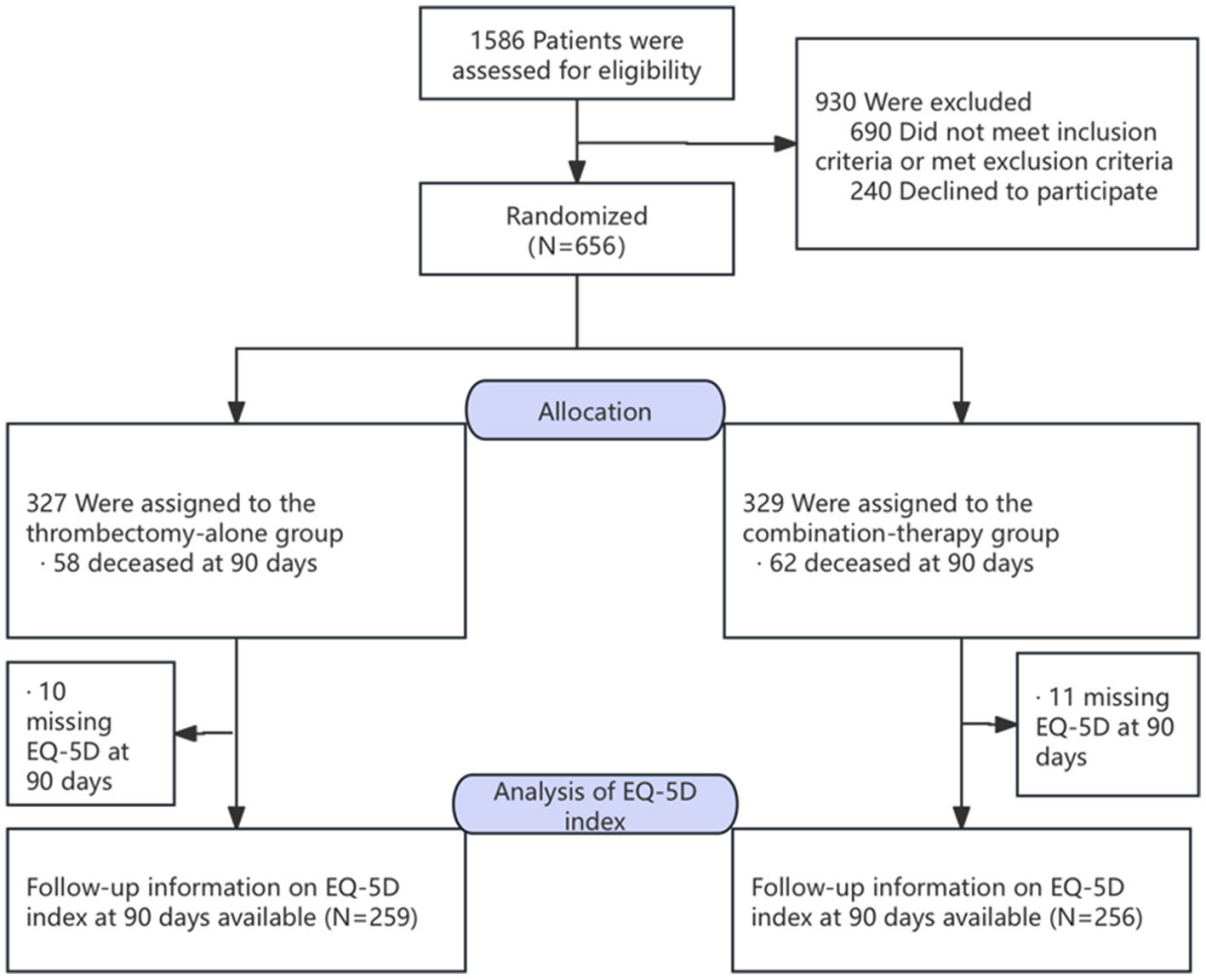
Study flow chart.

A total of 656 patients were included in this analysis, with 327 (49.85%) randomized to direct EVT and 329 (50.15%) to bridging therapy. Baseline characteristics have been reported previously in the primary DIRECT-MT publication and were well balanced between treatment groups. One hundred and twenty patients (18.29%) died and 21 patients (3.20%) were lost to follow-up during the observation period. Among the remaining 515 patients, 259 (50.29%) were assigned to the thrombectomy-alone group and 256 (49.71%) were assigned to the combination-therapy group. More details could refer to the flowchart in Figure 1. As shown in Table 1, the majority of clinical characteristics had no significant differences between patients with and without available EQ-5D data, except for baseline NIHSS score, and medical history of hypertension and diabetes.

**Table 1:** Demographic and clinical characteristics of patients at baseline.

Variable	Patients with EQ-5D *n* = 515	Patients without EQ-5D *n* = 21	*P* ^#^	Dead patients *n* = 120
Mean age ± SD, years	67.1 ± 12.0	68.1 ± 12.3	0.731	71.0 (62.5–78.5)
Gender, *n* (%)				
Male	294 (57.1)	11 (52.4)	0.669	65 (54.2)
Female	221 (42.9)	10 (47.6)		55 (45.8)
Median systolic blood pressure at hospital arrival, mm Hg	146 (130–163)	142 (139–170)	0.416	149.0 (132.5–169.0)
Median diastolic blood pressure at hospital arrival, mm Hg	83 (76–95)	89 (80–96)	0.202	83.0 (78.5–92.5)
Medical history, *n* (%)				
Previous ischemic stroke	73 (14.2)	2 (9.5)	0.778^*^	15 (12.5)
Atrial fibrillation	222 (43.1)	12 (57.1)	0.204	67 (55.8)
Hypertension	303 (58.8)	17 (81.0)	**0.043**	74 (61.7)
Diabetes mellitus	80 (15.5)	8 (38.1)	**0.015** ^*^	36 (30.0)
Median baseline NIHSS score (severity of neurological deficit) (IQR)^a^	16 (12–20)	19 (18–24)	**0.002**	21 (14.5–24.5)
Median ASPECTS at admission (IQR)^f^	9 (7–10)	8 (6–10)	0.332	8 (6–9)
Use of intravenous thrombolysis, *n* (%)	256 (49.7)	10 (47.6)	0.851	57 (47.5)
Median duration (IQR)—min				
From stroke onset to randomization	174.5 (124.5–209.5)	233.0 (203.0–239.5)	0.250	179.0 (142.0–219.5)
From randomization to start of alteplase^**^	7.0 (4.0–12.0)	8.5 (4.5–13.0)	0.158	7.0 (3.0–12.0)
From randomization to groin puncture^++^	36.0 (23.0–50.5)	44.0 (36.5–64.5)	0.215	33.0 (21.0–49.5)
From randomization to revascularization	88.0 (62.0–119.0)	106.0 (75.0–184.0)	0.121	105 (66–136)
From hospital admission to intravenous alteplase	59.0 (44.0–77.0)	53.5 (44.5–59.0)	0.590	62.0 (49.5–79.5)
From hospital admission to groin puncture	86.0 (69.0–115.0)	91.5 (79.0–129.0)	0.347	86.0 (74.5–106.5)
Median glucose level at hospital arrival (IQR), mmol/liter^b^	6.8 (5.8–8.2)	8.3 (5.6–9.1)	0.297	7.7 (6.5–10.9)
Hemisphere CTA, *n* (%)				
Left	227 (44.1)	11 (52.4)	0.453	55 (45.8)
Right	288 (55.9)	10 (47.6)		65 (54.2)
Location of intracranial artery occlusion, *n*(%)^c^				
Intracranial ICA	158 (31.5)	7 (35.0)	0.818	61 (51.7)
M1 middle cerebral artery segment	285 (56.8)	10 (50.0)		44 (37.3)
M2 middle cerebral artery segment	59 (11.8)	3 (15.0)		13 (11.0)
Cause of stroke, *n* (%)^d^				
Cardioembolism	217 (42.1)	12 (57.1)	0.388^†^	61 (50.8)
Intracranial atherosclerosis	32 (6.2)	2 (9.5)		11 (9.2)
Ipsilateral extracranial ICA obstruction	54 (10.5)	1 (4.8)		8 (6.7)
Uncertain	212 (41.2)	6 (28.6)		40 (33.3)
Reperfusion before intervention (eTICI) DSA, *n* (%)^e^				
0	19 (3.9)	3 (15.0)	0.391^†^	13 (11.3)
1	1 (0.2)	0 (0.0)		3 (2.6)
2a	50 (10.3)	2 (10.0)		21 (18.3)
2b	147 (30.2)	6 (30.0)		36 (31.3)
2c/3	270 (55.4)	9 (45.0)		42 (36.5)
Median NIHSS at 5–7 days or discharge (IQR)	5 (1–12)	13 (8–24)		42 (25–42)
Treatment, *n* (%)				
Direct EVT	259 (50.3)	10 (47.6)	0.810	58 (48.3)
Combination of IVT and EVT	256 (49.7)	11 (52.4)		62 (51.7)
Pre-stroke modified Rankin scale score, *n* (%)^g^				
0	477 (92.6)	18 (85.7)	0.168^†^	110 (91.7)
1	26 (5.0)	3 (14.3)		9 (7.5)
2	12 (2.3)	0 (0.0)		1 (0.8)

EQ-5D, EuroQol-5 dimension interment for measuring quality of life; SD, standard deviation; IQR interquartile range; NIHSS national institutes of health stroke scale; eTICI expanded treatment in cerebral infarction; DSA, digital subtraction angiography; ICA, internal carotid artery; CTA, computed tomography angiography.

^#^*P*-value testing differences in the distribution of baseline characteristics between patients with and without EQ-5D follow-up data.

^*^Yates's correction for continuity of Chi-square test due to one or more theoretical frequency < 5.

^†^Fisher's exact test due to more than 25% theoretical frequency < 5.

^a^Scores on the NIHSS range from 0 to 42, with higher scores indicating more severe neurological deficits.

^b^Data available for 118 deceased patients.

^c^Data available for 502 patients with EQ-5D, 20 patients without EQ-5D and 118 deceased patients.

^d^The cause of stroke was assessed according to the medical history, clinical features, and results on digital subtraction angiography.

^e^Data available for 487 patients with EQ-5D, 20 patients without EQ-5D and 115 deceased patients.

^f^The Alberta stroke program early computed tomography score (ASPECTS) is a measure of the extent of early cerebral ischemia. Scores ranges from 0 to 10, with higher scores indicating fewer early ischemic changes. Shown are values as assessed by the core laboratory.

^g^Scores on the modified Rankin scale of functional recovery range from 0 (no symptoms) to 6 (death). A score of 2 or less indicates functional independence. The modified Rankin scale score before stroke onset was assessed by the treating physician with the use of information obtained from patients (if possible) or their family members. Only patients with a modified Rankin scale score of 0–2 were included in the trial.

^**^For the time from randomization to the start of alteplase administration, data available for 256 patients with EQ-5D, 10 patients without EQ-5D.

^++^For the time from randomization to groin puncture, data available for 499 patients with EQ-5D, 20 patients without EQ-5D.

### EQ-5D index across BI and mRS subgroups

At 90 days, the overall median EQ-5D index with deaths excluded was similar between direct EVT group and bridging therapy group (0.84 vs. 0.85). Stratified analyses revealed heterogeneity by functional outcome subgroups (as shown in Table 2). Across baseline NIHSS strata, EQ-5D index decreased with increasing stroke severity, with broadly similar medians between treatment groups in NIHSS 0–15 and 16–20 and very low values in NIHSS 21–42 (Table 2). Among patients with BI at 90 days of 61–90 scores, median EQ-5D index was higher in the direct EVT group compared with bridging therapy group (0.74 vs 0.62). Conversely, among those with BI at 90 days of 91–100, bridging patients had slightly higher EQ-5D (1.00 vs 0.93). By mRS, patients with moderate disability (mRS 3–5) had higher EQ-5D index after direct EVT group compared with bridging therapy group (0.54 vs 0.35), whereas those with good outcome (mRS 0–2) showed comparable values (0.95 vs 1.00). Sensitivity analyses using imputation of missing values and death assigned as a value of 0 yielded similar results (Table 2).

**Table 2:** Median EQ-5D index (IQR) at 90 days by subgroups of baseline NIHSS and Barthel index and mRS at 90 days, and treatment for all analyses (death at 90 days assigned as 0, with imputation of missing values, and with deaths excluded).

Subgroup	Median EQ-5D index^#^	
	Endovascular thrombectomy	Alteplase with endovascular thrombectomy
Death assigned as 0		
Overall	0.74 (0.00–0.94)	0.65 (0.00–0.99)
Baseline NIHSS		
0–15	0.89 (0.52–1.00)	0.86 (0.17–1.00)
16–20	0.73 (0.00–0.90)	0.73 (0.00–1.00)
21–42	0.11 (0.00–0.63)	0.17 (0.00–0.91)
Barthel index at 90 days^##^		
0–20	0.03 (−0.01–0.12)	−0.01 (−0.08–0.10)
21–60	0.19 (0.02–0.33)	0.16 (0.05–0.27)
61–90	0.74 (0.58–0.85)	0.62 (0.44–0.77)
91–100	0.93 (0.85–1.00)	1.00 (0.90–1.00)
mRS at 90 days		
0–2	0.95 (0.89–1.00)	1.00 (0.94–1.00)
3–5	0.54 (0.11–0.80)	0.35 (0.06–0.77)
6	0.00 (0.00–0.00)	0.00 (0.00–0.00)
Death assigned as 0 and missing scores imputed^*^		
Overall	0.72 (0.00–0.94)	0.64 (0.00–0.96)
Baseline NIHSS		
0–15	0.89 (0.52–1.00)	0.85 (0.14–1.00)
16–20	0.73 (0.00–0.90)	0.64 (0.00–0.99)
21–42	0.06 (0.00–0.62)	0.17 (0.00–0.90)
Barthel index at 90 days^##^		
0–20	0.05 (−0.01–0.14)	0.03 (−0.08–0.13)
21–60	0.19 (0.02–0.33)	0.16 (0.05–0.28)
61–90	0.75 (0.58–0.85)	0.62 (0.44–0.77)
91–100	0.93 (0.85–1.00)	1.00 (0.90–1.00)
mRS at 90 days		
0–2	0.95 (0.89–1.00)	1.00 (0.94–1.00)
3–5	0.53 (0.11–0.80)	0.39 (0.06–0.77)
6	0.00 (0.00–0.00)	0.00 (0.00–0.00)
Death excluded		
Overall	0.84 (0.50–0.95)	0.85 (0.27–1.00)
Baseline NIHSS		
0–15	0.89 (0.52–1.00)	0.85 (0.14–1.00)
16–20	0.73 (0.00–0.90)	0.64 (0.00–0.99)
21–42	0.06 (0.00–0.62)	0.17 (0.00–0.90)
Barthel index at 90 days^##^		
0–20	0.03 (−0.01–0.12)	−0.01 (−0.08–0.10)
21–60	0.19 (0.02–0.33)	0.16 (0.05–0.27)
61–90	0.74 (0.57–0.85)	0.62 (0.44–0.77)
91–100	0.93 (0.85–1.00)	1.00 (0.90–1.00)
mRS at 90 days		
0–2	0.95 (0.89–1.00)	1.00 (0.94–1.00)
3–5	0.54 (0.11–0.80)	0.35 (0.06–0.77)
6	NA	NA

NIHSS National Institutes of Health stroke scale; values are presented as median (interquartile range, IQR). Deaths were assigned an EQ-5D index of 0 in the primary analysis.

^*^In the sensitivity analyses, deaths were assigned 0 and missing values imputed with the multiple imputation with truncated regression, or deaths were excluded entirely.

^#^Scores on the EQ-5D index range from −0.39 [worst] to 1.00 [best] with higher values indicating a better quality of life.

^##^The Barthel index score [range, 0 (severe disability) to 100 (no disability)].

### Regression analyses of treatment-by-subgroup interactions

#### Effect modification by baseline stroke severity (NIHSS)

**Figure 2:**
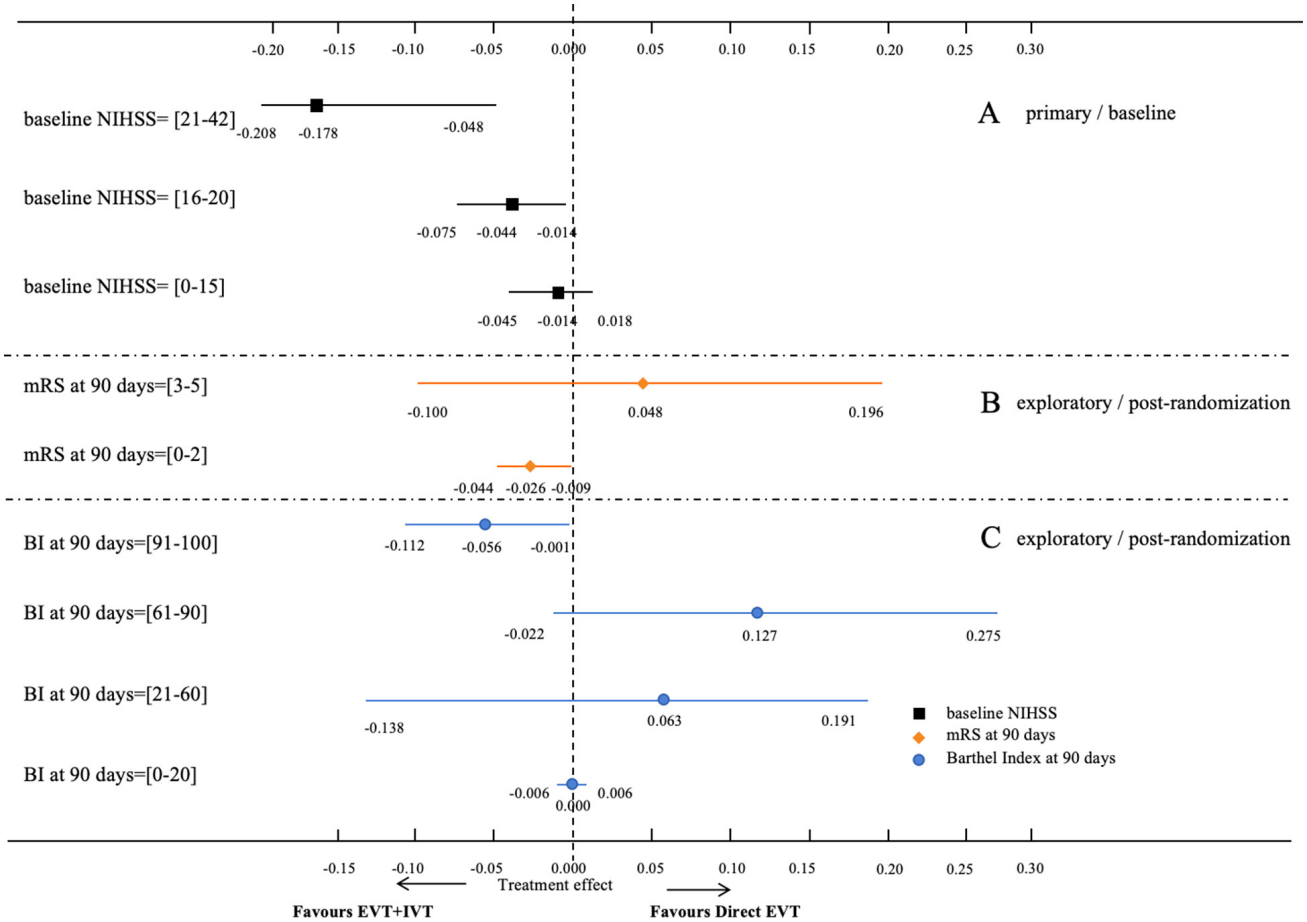
Forest plots of the estimated treatment effect of direct EVT vs. Alteplase with EVT on median EQ-5D index at 90 days, based on quantile regression models. Results are presented by **(A)** baseline NIHSS categories (primary analysis; pre-randomization effect modification), and by exploratory post-randomization functional strata defined at 90 days: **(B)** mRS (0–2 vs. 3–5) and **(C)** Barthel index (BI) (0–20, 21–60, 61–90, 91–100). Points represent regression coefficients and whiskers indicate 95% confidence intervals. Positive values indicate higher EQ-5D scores with direct EVT.

Baseline stroke severity significantly modified the association between treatment strategy and EQ-5D outcomes (*P* for interaction=0.038). As Figure 2A showed, in analyses stratified by baseline stroke severity (NIHSS), the estimated treatment difference in EQ-5D (direct EVT vs bridging therapy) was not significant in the NIHSS 0–15 subgroup (−0.014, 95% CI −0.045 to 0.018; *P* = 0.398). However, bridging therapy was associated with higher EQ-5D indexes compared with direct EVT in moderate-to-severe (NIHSS 16–20) (−0.044, 95% CI −0.075 to −0.014; *P* = 0.004) and severe (NIHSS 21–42) (−0.278, 95% CI −0.508 to −0.048; *P* = 0.018) patients.

#### Exploratory post-randomization stratifications

To describe how HRQoL differences manifest across achieved functional states at follow-up, we additionally conducted exploratory analyses stratified by 90-day BI and mRS (Figures 2B, C). These post-treatments stratified analyses were descriptive and not intended to support baseline treatment selection; accordingly, findings are interpreted as hypothesis-generating. For BI, bridging therapy was associated with higher EQ-5D indexes among the patients with slight dependence and independence (BI 91–100) (coefficient −0.056; 95% CI −0.112 to −0.001; *P*_int_ = 0.025). However, direct EVT showed a trend toward higher EQ-5D indexes among groups with moderate to severe dependence, while they were not statically significant. For mRS, direct EVT was associated with lower EQ-5D indexes among patients with favorable functional outcome (mRS 0–2) (coefficient −0.026; 95% CI −0.044 to −0.009; *P*_int_ = 0.004), while bridging therapy was associated with higher EQ-5D indexes among those with unfavorable functional outcome (mRS 3–5) (coefficient 0.048; 95% CI −0.100 to +0.196; *P*_int_ = 0.525).

In the sensitivity analysis imputing missing EQ-5D index values, the results were very similar to the main analysis (Supplemental Figure S1). In the second sensitivity analysis excluding deaths, the interaction of treatment with BI at 90 days was significant for group BI 61–90 scores (*P*_int_ = 0.036 for BI group 61–90; *P*_int_ = 0.040 for global interaction), which group was associated with higher EQ-5D indexes with direct EVT [0.11 (95% CI 0.01–0.21); Supplemental Figure S2]. The subgroup of severe dependence (BI 0-20) showed higher EQ-5D indexes compared to the main analysis. All observed relationships were similar when regressing on the 25th or 75th percentile of EQ-5D indexes, implying that this observed effect modification was a distributional effect across the EQ-5D scores.

### EQ-5D-5L domains

**Figure 3:**
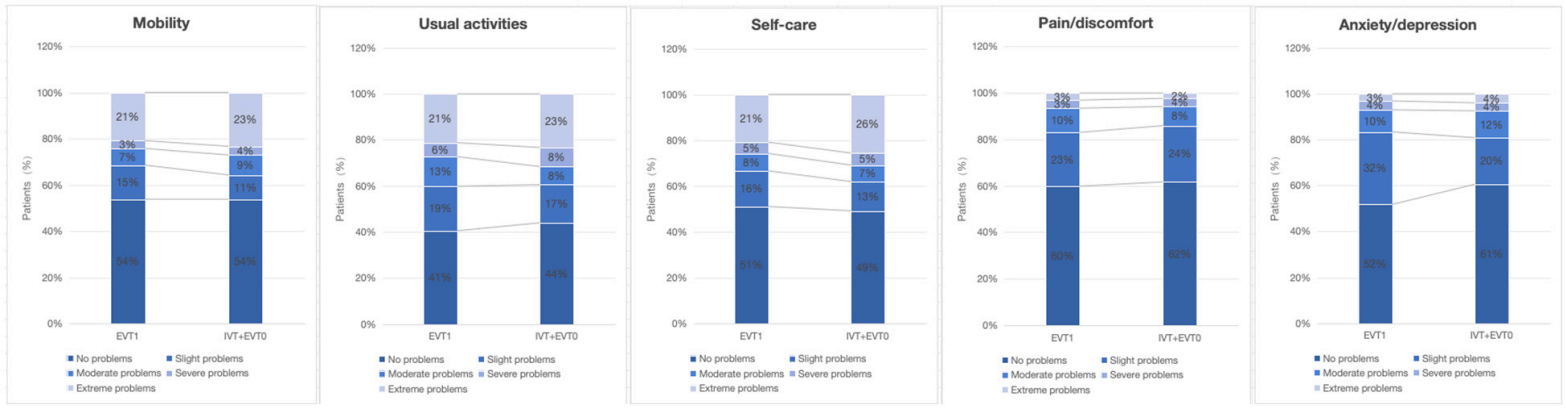
Percent of patients within each domain of EuroQol-5D [level 1 (no problems), 2 (slight problems), 3 (moderate problems), 4 (severe problems), or 5 (extreme problems)], stratified by thrombectomy-alone group and combination-therapy group.

**Figure 4:**
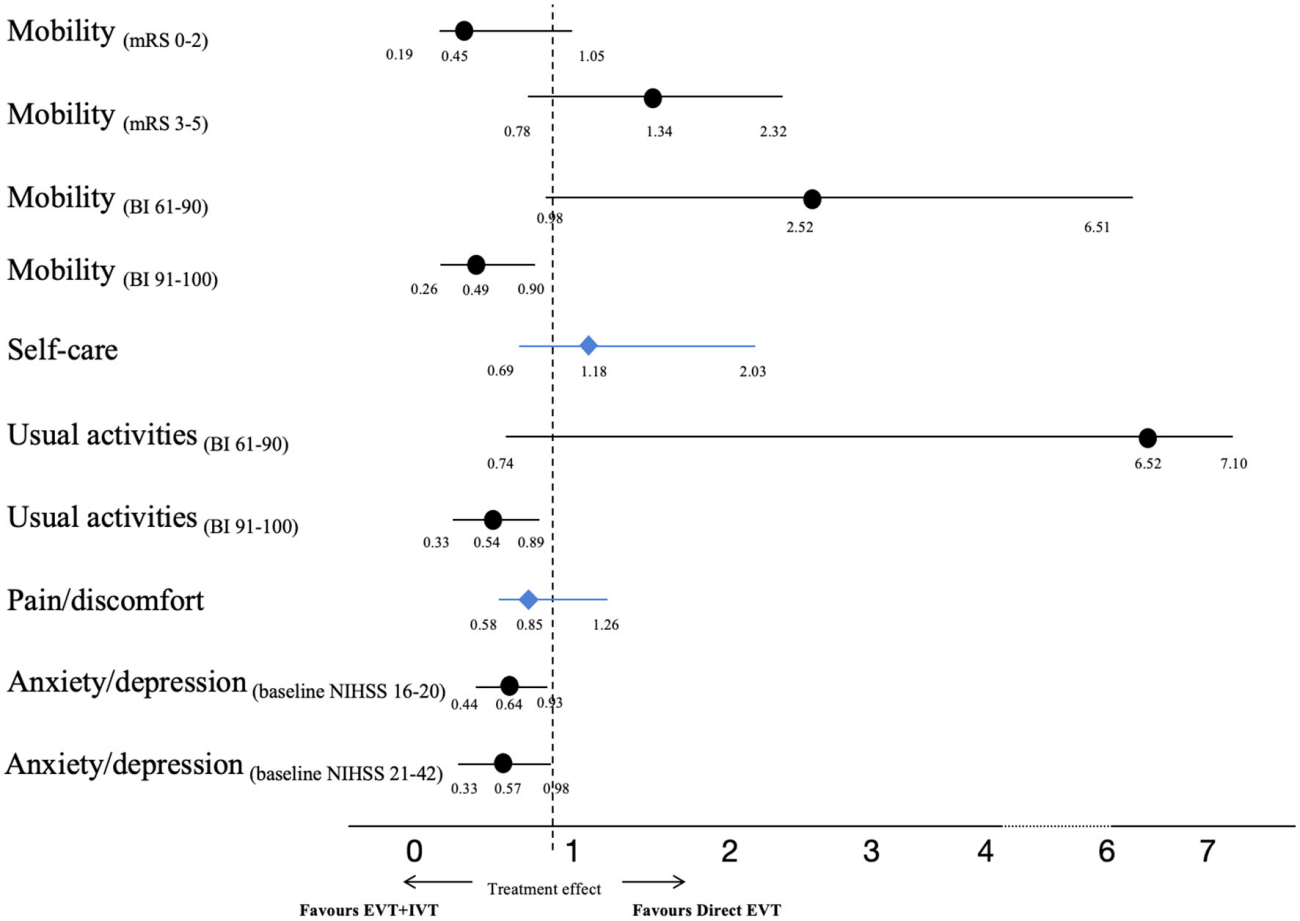
Adjusted odds of achieving symptom-free status with direct EVT in each domain of EuroQol-5D. Dimensions are stratified by baseline NIHSS, BI and mRS at 90 days only (with Black circles) when *P* value for interaction is significant (*P* < 0.05). The blue lines indicate the overall effect of treatment on the EQ-5D domains. For better visualization, categories of baseline NIHSS 0–15, BI 0–20 and BI 21–60 are omitted (*P* > 0.05).

Across the five EQ-5D-5L dimensions, the proportions of patients reporting no symptom, slight symptoms, moderate problems, severe problems or extreme problems are shown in Figure 3. Overall, Mobility and Self-care limitations remained frequent, whereas Pain/discomfort and Anxiety/depression were less common. Compared with bridging therapy group, direct EVT patients reported fewer problems in Mobility and Self-care, but a slightly higher proportion reported symptoms in Pain/discomfort and Anxiety/depression domains. In adjusted logistic regression for Mobility, there was a significant interaction with BI at 90 days (*P*_int_ = 0.018 for global interaction), whereby only BI ranging from 91–100 had a lower odd of symptom-free status of Mobility with direct EVT [aOR 0.49 (95% CI, 0.26–0.90); *P* = 0.022]. Although only Mobility dimension and mRS initially were detected an overall interaction (*P*_int_ = 0.030 for global interaction), the interaction effects at each level of mRS were not statistically significant in further testing (Figure 4). Unfavorable disability (mRS 3–5) had a higher odd of symptom-free status with direct EVT [aOR 1.34 (95% CI, 0.78–2.32); *P* = 0.291]. There was a significant interaction with BI at 90 days (*P*_int_ = 0.032 for global interaction) for Usual activities, whereby only patients with slight dependence and independence status (BI 91–100) had a lower odd of symptom-free status with direct EVT [aOR 0.54 (95% CI, 0.33–0.89); *P* = 0.016]. There was a significant interaction with baseline NIHSS (*P*_int_ = 0.018 for global interaction) for Anxiety/depression. Moderate-to-severe (baseline NIHSS 16–20) and severe (baseline NIHSS 21–42) patients both had a lower odd of symptom-free status with direct EVT (both *P* < 0.05, Figure 4). There were no significant functional status interactions for Self-care and Pain/discomfort domains. However, direct EVT was associated with a higher odd of being symptom-free with Self-care [aOR, 1.18 (95% CI, 0.69–2.03); *P* = 0.543], but not Pain/discomfort [aOR, 0.85 (95% CI, 0.58–1.26); *P* = 0.419].

## Discussion

In this secondary analysis of the DIRECT-MT trial, overall HRQoL measured by the EQ-5D index at 90 days was broadly similar between patients randomized to direct EVT and those randomized to bridging therapy. Despite comparable overall distributions, we observed heterogeneity in EQ-5D across clinically relevant strata. Importantly, baseline stroke severity (NIHSS), a pre-randomization measure available at the time of treatment decision-making, significantly modified the association between treatment strategy and EQ-5D, with bridging therapy showing higher EQ-5D than direct EVT among patients with more severe baseline deficits (NIHSS 16–20 and 21–42). These findings add a patient-centered perspective to the parent trial results and highlight that treatment strategies may have differential implications for perceived health status across baseline severity.

Importantly, these results should not be interpreted as proposing HRQoL-guided individualized reperfusion strategies, but rather as descriptive evidence that patient-perceived outcomes may differ across baseline severity even when traditional functional outcomes are similar. Our findings extend the primary DIRECT-MT results, which demonstrated non-inferiority of EVT alone versus bridging therapy in terms of mRS-defined functional independence (6). Similar to our study, other recent trials (e.g., DEVT, SKIP, MR CLEAN-NO IV) reported no consistent advantage of bridging therapy for functional outcomes (18–23). However, prior studies have primarily focused on mRS outcomes. By incorporating EQ-5D, our analysis expands the evaluation of treatment efficacy to encompass HRQoL, an aspect increasingly recognized as critical for stroke survivors (24, 25). Rather than aiming to alter current guideline-recommended workflows, our results provide complementary, patient-centered information that may help contextualize recovery experiences following reperfusion therapy.

The finding that baseline NIHSS modified the HRQoL impact of treatment strategy is clinically relevant because it anchors heterogeneity in a baseline characteristic rather than post-treatment outcomes. The observed pattern—bridging therapy associated with higher EQ-5D among patients with more severe baseline stroke—may reflect several mechanisms (22, 25). Alteplase administered before thrombectomy could facilitate early or distal reperfusion, improve microvascular perfusion, or augment collateral flow, which may translate into differences in perceived recovery that are not fully captured by global disability scores (24). Conversely, any potential procedural delays or bleeding risks associated with alteplase might attenuate HRQoL in some patients. These mechanistic explanations remain speculative, and given real-world constraints—particularly in hub-and-spoke systems and resource-limited settings where eligibility for bridging therapy may be lower—our findings should be interpreted as hypothesis-generating.

In addition to baseline severity, we conducted stratified analyses by 90-day BI and mRS to elucidate where along the functional recovery continuum differences in HRQoL between reperfusion strategies become most apparent. Although BI, mRS, and EQ-5D were assessed at the same follow-up time point, these post-randomization stratifications provide important descriptive insight into how patient-perceived health status aligns with achieved functional outcomes under different treatment strategies. Notably, treatment-related differences in EQ-5D were not uniform across BI and mRS strata, suggesting that HRQoL may be particularly sensitive to treatment effects in specific recovery states, such as near-complete independence or favorable functional outcome, where subtle residual symptoms and patient perceptions are more likely to influence quality-of-life assessments. While subgroup estimates varied across analytic approaches, the overall pattern highlights meaningful heterogeneity in HRQoL expression across functional states and underscores the complementary role of HRQoL in interpreting post-stroke recovery beyond traditional disability scales.

Dimension-level analyses further clarified how patient experiences may differ even when overall EQ-5D index values are similar. Mobility and self-care limitations remained common, consistent with persistent physical disability after large-vessel occlusion despite successful reperfusion (16, 26–28). Across dimensions, we observed interaction signals with BI or mRS for selected domains, suggesting that the relationship between treatment strategy and “symptom-free” status may differ according to functional outcome strata. For Pain/discomfort, EVT-alone patients tended to report more symptoms, which may reflect post-stroke pain syndromes or recovery-related complications (29). Notably, baseline NIHSS interacted with treatment assignment specifically for the Anxiety/depression dimension, with more severe baseline stroke associated with lower odds of being symptom-free after direct EVT compared with bridging therapy. This finding highlights emotional health as a potentially important contributor to HRQoL differences and supports the incorporation of psychological assessment and support into post-stroke care pathways (30–32). Given the multiple comparisons performed, these domain-specific findings should be interpreted cautiously and warrant external validation.

## Strengths and limitations

The strengths of this study include its randomized design, large sample size, and systematic use of a validated HRQoL instrument, providing robust evidence on patient-centered outcomes after reperfusion therapy. The prespecified subgroup analyses by BI and mRS further enhance the clinical interpretability of our findings. Nevertheless, several limitations should be acknowledged. First, this is a secondary analysis, and causality cannot be definitively inferred. Second, missing HRQoL data and deaths were addressed through imputation and sensitivity analyses, but residual bias cannot be excluded. Third, subgroup analyses based on 90-day BI and mRS are post-randomization and may be subject to post-treatment stratification; therefore, these findings should be exploratory and interpreted as hypothesis-generating. Finally, long-term HRQoL beyond 90 days was not assessed, and further research is needed to examine whether treatment-related differences persist over time.

## Conclusions

In summary, direct EVT and bridging therapy produced comparable overall HRQoL at 90 days, but treatment effects on HRQoL varied by baseline stroke severity, with bridging therapy associated with higher EQ-5D among patients with more severe baseline NIHSS. Exploratory post-treatment stratified analyses by BI and mRS and dimension-level findings provide additional context regarding how HRQoL differences may present across functional outcomes, but they should be interpreted cautiously. These results support continued evaluation of patient-centered outcomes in reperfusion trials and motivate future prospective studies to validate baseline severity-guided approaches for optimizing HRQoL after large-vessel occlusion.

## Data Availability

The raw data supporting the conclusions of this article will be made available by the authors, without undue reservation.
