# Supplementary material for: Health-related quality of life after direct endovascular thrombectomy and bridging therapy: findings of DIRECT-MT trial
Source: Front Neurol. 2026 May 25;17:1768325. doi: 10.3389/fneur.2026.1768325 (PMC13243117; doi:10.3389/fneur.2026.1768325)
Supplement: Supplementary file 1 [file Supplementary_File_1.docx]

**Supplementary Material**

**Figure S1 in the Data Supplement (imputing missing EQ-5D index values)**


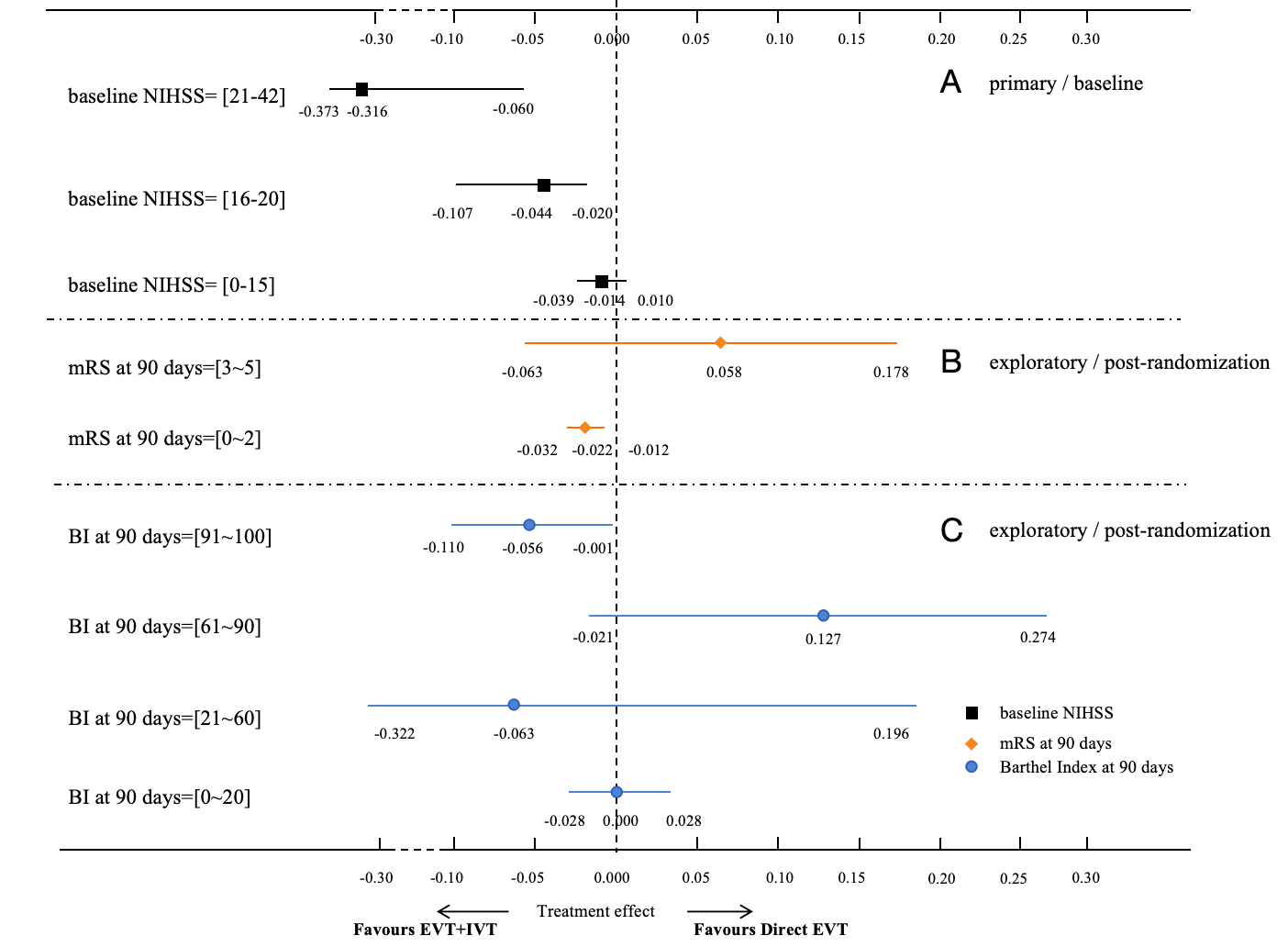
(Ⅰ)


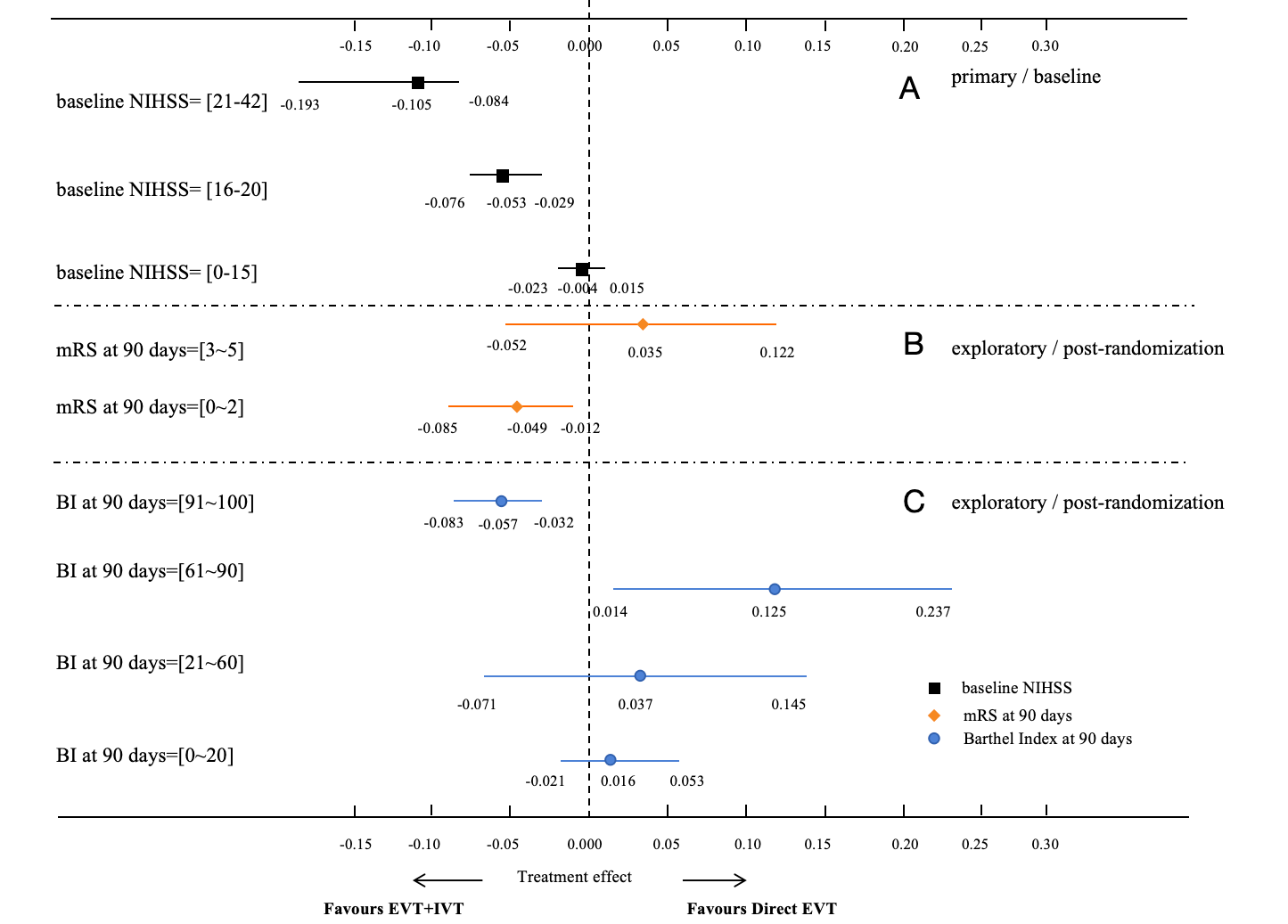
(Ⅱ)

**Figure S1. Forest plots of the estimated treatment effect of direct EVT versus bridging therapy (alteplase plus EVT) on median EQ-5D index at 90 days, based on quantile regression models (imputing missing EQ-5D index values).**

Results are presented by (A) baseline NIHSS categories (primary analysis; pre-randomization effect modification), and by exploratory post-randomization functional strata defined at 90 days: (B) mRS (0–2 vs 3–5) and (C) Barthel Index (BI) (0–20, 21–60, 61–90, 91–100). Points represent regression coefficients and whiskers indicate 95% confidence intervals. Positive values indicate higher EQ-5D scores with direct EVT. Predicted EQ-5D values of control and direct EVT groups are shown for primary analysis (Ⅰ) and when excluding deaths (Ⅱ).


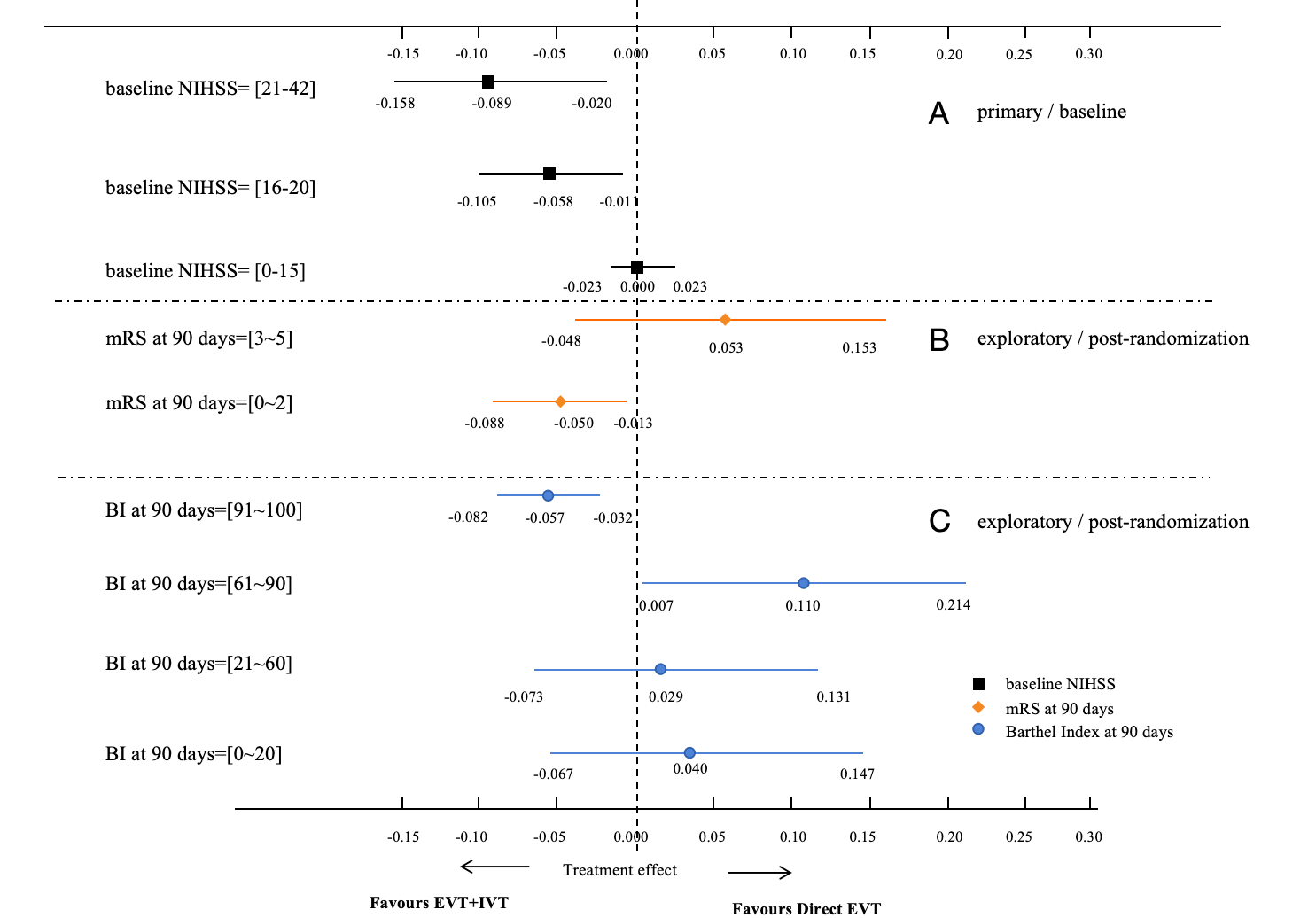


**Figure S2. Forest plots of the estimated treatment effect of direct EVT versus bridging therapy (alteplase plus EVT) on median EQ-5D index at 90 days, based on quantile regression models (sensitivity analysis of excluding deaths).**

Results are presented by (A) baseline NIHSS categories (primary analysis; pre-randomization effect modification), and by exploratory post-randomization functional strata defined at 90 days: (B) mRS (0–2 vs 3–5) and (C) Barthel Index (BI) (0–20, 21–60, 61–90, 91–100). Points represent regression coefficients and whiskers indicate 95% confidence intervals. Positive values indicate higher EQ-5D scores with direct EVT.
